# Metastatic giant basal cell carcinoma: a case report

**DOI:** 10.11604/pamj.2016.24.157.9233

**Published:** 2016-06-22

**Authors:** Khadija Bellahammou, Asmaa Lakhdissi, Othman Akkar, Fadoua Rais, Benhmidou Naoual, Ibrahim Elghissassi, Hind M’rabti, Hassan Errihani

**Affiliations:** 1 Department of Medical Oncology, National Institute of Oncology, Rabat, Morocco; 2 Department of Radiotherapy, National Institute of Oncology, Rabat, Morocco

**Keywords:** Giant basal cell carcinoma, metastasis, prognosis

## Abstract

Basal cell carcinoma is the most common skin cancer, characterised by a slow growing behavior, metastasis are extremely rare, and it occurs in less than 0, 1% of all cases. Giant basal cell carcinoma is a rare form of basal cell carcinoma, more aggressive and defined as a tumor measuring more than 5 cm at its largest diameter. Only 1% of all basal cell carcinoma develops to a giant basal cell carcinoma, resulting of patient's negligence. Giant basal cell carcinoma is associated with higher potential of metastasis and even death, compared to ordinary basal cell carcinoma. We report a case of giant basal cell carcinoma metastaticin lung occurring in a 79 years old male patient, with a fatal evolution after one course of systemic chemotherapy. Giant basal cell carcinoma is a very rare entity, early detection of these tumors could prevent metastasis occurrence and improve the prognosis of this malignancy.

## Introduction

Basal cell carcinoma (BCC) is the most common malignant skin tumor in the world with 750,000 cases reported annually in the United States alone [1, 2]; it is more common in males than females. BCC is a slow growing tumor; metastasis are extremely rare and reported in less than 0.1 %of cases [3]. The giant basal cell carcinoma, defined as a lesion with more than 5 cm at its largest diameter, is very rare and presents less than 1% of all basal cell carcinoma [3–5]. Their potential of metastasis is higher than ordinary basal cell carcinoma and the prognosis is very poor. Due to rarity of metastatic disease, all cases published in literature are limited to retrospective studies and cases reports. In this article, we present a case of giant basal cell carcinoma occurring in a 79 years old male patient metastaticin lungs with a fatal evolution.

## Patient and observation

**Figure 1:**
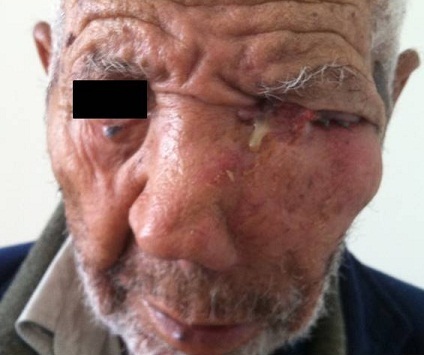
Ulcerated lesion extending to the left orbit

**Figure 2:**
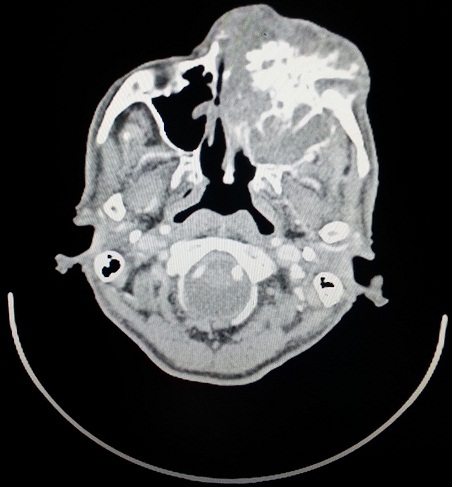
Facial computed tomography showing a large enhancing mass of the left maxillary sinus

**Figure 3:**
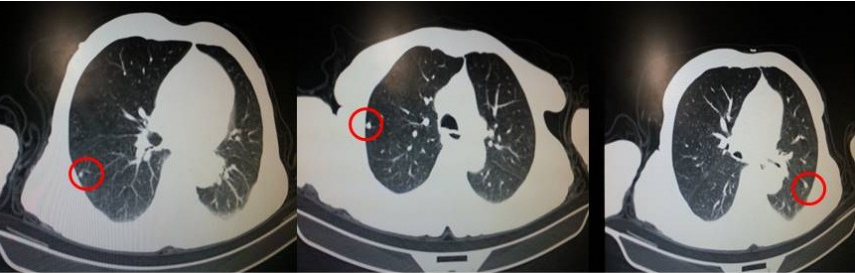
Thoracic computed tomography showing bilateral suspicious lung nodules

A 79 year-old male presented to our institution complaining of a painful ulcerated skin lesion localized in the left lower eyelid. The patient's history found no comorbidities. On Physical examination, an ulcerated lesion extending to the left orbite was noted (Figure 1). A facial neck and chest computed tomography showed a large enhancing mass of the left maxillary sinus measuring 60×65mm with bilateral suspicious lung nodules (Figure 2, Figure 3). A biopsy of the mass was performed, histopathological examination revealed an infiltrative basal cell carcinoma. A diagnosis of a giant basal cell carcinoma metastatic to the lungs was established; the patient had an ECOG performance status of 2, a blood workup showed he had normal renal function. Platinum based chemotherapy was decided. He received only one course of 5FU-ceplatin then decided to interrupt the treatment. Unfortunately he died one month after the beginning of treatment.

## Discussion

Basal cell carcinoma is the most common malignant skin tumor and isusually characterized by a slow growth. Despite its high incidence, metastatic events are exceedingly rare. The reported frequency of metastatic dissemination is estimated to be less than 0.1% [3]. Risk factors include: Chronic sun exposure, radiation, light skin color, Immune-suppression, Exposure to arsenic, certain hereditary disease including Gorlin-Goltz syndrome and Xerodermapigmentosum, advanced age and male sex [6]. The clinical appearances and morphology are diverse, including micronodular, nodular, infiltrative, superficial, sclerosing, morpheaform, keratotic cystic and pigmented variants [7].

The giant basal cell carcinoma is a rare form of basal cell carcinoma normally defined as a basal cell carcinoma with more than 5 cm at its largest diameter. Only 1% of basal cell carcinoma develops to a giant basal cell carcinoma, due to long duration and patient's negligence in most cases [4]. Giant basal cell carcinoma has been associated with higher incidence of metastasis compared to the ordinary basal cell carcinoma [3, 4] typically to lymph nodes, bone, and lung. The prognosis is poor and the median survival in metastatic cases is around 8 months [8].

Sites of predilection for basal cell carcinoma are head and neck; giant basal cell carcinomas were most likely to occur on the back, face, and upper extremities. The pathogenesis of giant basal cell carcinoma is linked to a mutation of the PTCH gene, mapped to the q22.3 locus of chromosome. This gene has a role in tumor suppression by inhibiting a regulatory signaling cascade of the Sonic Hedgehog pathway [9].

Treatment modalities include simple excision, electrodessication, curettage, cryotherapy, Mohs Micrographic surgery, topical treatment (imiquimod), photodynamic therapy, radiotherapy and chemotherapy. Chemotherapy for giant basal cell carcinoma can be local and systemic. Systemic treatment is considered for inoperable and metastatic lesions. Due to the rarity of metastatic cases, the literature on chemotherapy for basal cell carcinoma is limited to cases reports. Drugs used for systemic therapy include methotrexate, bleomycin, vincristine, 5-FU, cyclophosphamide, dactinomycin and toyomycin, platinum and taxanes [9]. Vismodegib, a Hedgehog pathway inhibitor is now approved for the treatment of metastatic or locally advanced BCC that has recurred after surgery or which is incurable with surgery or radiation, [10].

## Conclusion

Although only 1% of basal cell carcinoma develops into giant basal cell carcinoma, early detection of these tumors by physicians or patients could prevent tumor extension and reduce the risk of metastasis which leads to improve the prognosis of such rare entity.
